# SCORE2 cardiovascular risk score for cognitive impairment in women aged 40 years and above: a pragmatic triage signal in primary care — a single-center family medicine feasibility study with machine learning support

**DOI:** 10.3389/fmed.2026.1884887

**Published:** 2026-08-19

**Authors:** Eda Çelik Güzel, Murat Güzel

**Affiliations:** 1 Department of Family Medicine, Faculty of Medicine, Tekirdağ Namık Kemal University, Tekirdağ, Türkiye; 2 Department of Computer Technology, Mucur Vocational School, Kırşehir Ahi Evran University, Mucur, Kırşehir, Türkiye

**Keywords:** cardiovascular risk, cognitive impairment, glucose tolerance, hyperlipidemia, hypertension, machine learning, primary care, SCORE2 triage

## Abstract

**Background:**

Screening-defined cognitive impairment (CI; MMSE ≤23) in midlife women is under-recognized in primary care. SCORE2 is a 10-year cardiovascular disease risk score already routinely calculated in many primary-care settings. We evaluated whether SCORE2 provides a clinically useful triage signal for CI; secondarily, a parsimonious machine learning (ML) model was developed from routinely-collected data to support the SCORE2 signal.

**Methods:**

In this single-center, cross-sectional feasibility study, 89 women aged 40–68 years (52 hypertensive, 37 normotensive controls) were analyzed. CI was defined as a Mini-Mental State Examination (MMSE) total score ≤23 (validated Turkish cut-off). The association between SCORE2 (high-risk-region coefficients) and CI was assessed by χ^2^ for trend, Spearman correlation, logistic regression, and ROC analysis. A parsimonious 5-variable ML model (age, BMI, systolic blood pressure, HT duration, folate) was evaluated by 10-times-repeated 5-fold cross-validation with bootstrap optimism correction, calibration, and Decision Curve Analysis. Reporting followed TRIPOD+AI 2024.

**Results:**

SCORE2 showed a clear dose–response association with CI: prevalence rose from 21.2% (Low) to 50.0% (Moderate), 62.5% (High), and 60.0% (Very High) (χ^2^ trend *p* = 0.006; Spearman *ρ* = 0.37, *p* < 0.001). Each 5% absolute SCORE2 increase conferred OR 3.65 (95% CI 1.67–7.98, *p* = 0.001); High vs. Low OR 6.21 (1.85–20.86, *p* = 0.003). SCORE2 alone discriminated CI with AUC 0.742 (0.62–0.85). Overall CI prevalence was 36.0% (32/89): 53.8% in hypertensives vs. 10.8% in controls (relative risk 4.98; NNS = 2.3). Prevalence rose monotonically across cardiometabolic strata (normotensive→Stage 2 HT: 11.1% → 60.6%; normal-BMI → obese: 13.8% → 59.0%; cumulative score 0 → 4: 7.7% → 66.7%; all *p* ≤ 0.002). The supportive parsimonious ML model achieved AUC 0.804 ± 0.103 (optimism-corrected 0.808; Brier 0.176; Hosmer-Lemeshow *p* = 0.397); incremental discrimination over SCORE2 was modest (ΔAUC +0.062). A bedside nomogram derived from the parsimonious model is provided as an exploratory tool pending external validation.

**Conclusion:**

SCORE2 — already routinely calculated in primary care — provides a clinically useful triage signal for cognitive impairment at no additional resource cost; women at high cardiovascular risk may benefit from formal cognitive testing. The parsimonious ML model mechanistically supports the SCORE2 signal. As a single-center feasibility study, multi-center prospective external validation is required before clinical deployment.

## 1 Introduction

Cognitive impairment (CI) imposes substantial individual, familial, and societal burden, with the worldwide population living with dementia projected to reach 153 million by 2050 (1). Hypertension (HT), obesity, dyslipidemia, and glucose intolerance are among the most consistently identified modifiable cardiovascular and metabolic risk factors for late-life cognitive decline (2–4). Mid-life HT in particular has been implicated in cerebral small-vessel disease, white-matter hyperintensities, and neurodegeneration (5–7). Despite this, routine cognitive screening in adult primary-care patients with HT remains uncommon.

SCORE2 is a standardized score, prospectively validated by the 2021 ESC guidelines, that predicts 10-year fatal and non-fatal cardiovascular events in individuals aged 40–69 years (8, 9). It is calculated as part of routine cardiovascular risk assessment in many primary-care settings. The components of SCORE2 (age, systolic blood pressure, total and HDL cholesterol, smoking status) are also established risk factors for cognitive decline; this overlap establishes the biological plausibility that SCORE2 may serve as a proxy marker for CI.

However, published studies that directly evaluate the association between SCORE2 and CI and position it as a tool for primary-care triage are limited. Most cognitive risk prediction models target older populations (≥60 years), large multi-center cohorts, or imaging-based phenotypes that are impractical in routine family medicine (10, 11). Pragmatic repurposing of an already-available primary-care tool for cognitive risk has high clinical translatability, as it requires no additional tests, equipment, or visit time.

Recent reporting standards for clinical prediction models (TRIPOD+AI, 2024) emphasize transparent reporting of sample size, calibration, and clinical utility alongside discrimination (12). The primary aim of this study is to characterize the association between SCORE2 and CI in women aged ≥40 years and evaluate SCORE2’s usability as a pragmatic triage signal for cognitive screening in primary care. The secondary aim is to develop a parsimonious ML model using routinely collected primary-care data to provide mechanistic support for this signal, and to present an exploratory bedside nomogram.

## 2 Materials and methods

### 2.1 Study design and setting

This was a single-center, cross-sectional, exploratory feasibility study conducted at the Department of Family Medicine, Tekirdağ Namık Kemal University. Reporting followed the TRIPOD+AI 2024 guideline (completed checklist provided as Supplementary Data Sheet 1) (12); and the STROBE recommendations for cross-sectional studies (Supplementary Data Sheet 3) (13). The study protocol was approved by the Tekirdağ Namık Kemal University Clinical Research Ethics Committee (Decision No. 2026.147.05.09; approval date: May 2026) and conducted in accordance with the Declaration of Helsinki. Written informed consent was obtained from all participants.

### 2.2 Participants

In this feasibility study, 89 women aged 40–68 years (52 hypertensive, 37 normotensive controls) were analyzed. The HT group comprised participants meeting the 2018 ESC/ESH criteria for hypertension (14) (office BP ≥ 140/90 mmHg on at least two visits, or current antihypertensive therapy). The control group comprised normotensive women (office BP < 140/90 mmHg with no history of antihypertensive use). Exclusion criteria were: prior diagnosis of dementia or major neurocognitive disorder; history of stroke or transient ischemic attack; severe psychiatric illness; current use of medications known to affect cognition; severe hepatic or renal failure; untreated overt thyroid disease; severe vitamin B12 deficiency under active replacement; and current pregnancy. All participants had completed at least primary education (≥5 years of formal schooling) and were literate; individuals with no formal schooling or who were illiterate were not enrolled.

### 2.3 Outcome definition

The primary outcome was cognitive impairment (CI), defined as a Mini-Mental State Examination (MMSE) (15) total score ≤23. This cut-off corresponds to the validated threshold for mild dementia established by Güngen et al. (16), yielding sensitivity 91% and specificity 95% against expert clinical diagnosis in 212 community-dwelling adults (16). The MMSE was administered face-to-face by a single trained family medicine resident blinded to laboratory results and blood pressure values at the time of testing. Throughout this manuscript, “cognitive impairment” refers operationally to MMSE ≤23 [the mild-dementia-suspect range per Güngen et al. (16)] and does not equate to a formal diagnosis of mild cognitive impairment (MCI) or dementia; the binary outcome should therefore be interpreted as a primary-care screening signal rather than a clinical diagnosis.

Because the binary outcome was directly derived from MMSE total score, MMSE total and its subdomain scores were excluded from the candidate predictor set to prevent target leakage.

### 2.4 Predictor variables

Eighteen routinely-collected primary-care variables formed the candidate predictor set: demographic and anthropometric (age, BMI, HT duration in years), lifestyle (smoking status, alcohol use, regular exercise), vital signs (office systolic and diastolic blood pressure), biochemistry (fasting plasma glucose, total cholesterol, triglycerides, HDL- and LDL-cholesterol, TSH, free T4, vitamin B12, folate), and a binary HT-status indicator. All laboratory analyses were performed in the institutional central laboratory using standardized commercial assays.

### 2.5 SCORE2 computation

Individualized 10-year cardiovascular disease risk was computed using the SCORE2 algorithm proposed by Hageman et al. (17) with recalibrated coefficients for the high-cardiovascular-risk region. SCORE2 inputs were age, smoking status, systolic blood pressure, total cholesterol, and HDL-cholesterol. Risk categories were defined per the 2021 ESC guidelines: Low (<2.5%), Moderate (2.5–5%), High (5–10%), and Very High (≥10%) (16).

### 2.6 Sample-size considerations

Following the framework of Riley et al. (18), the events-per-variable (EPV) ratio for a 5-predictor model (32/5 ≈ 6.4) approached the conventional minimum. The work was therefore explicitly framed as an exploratory feasibility study; SCORE2 was positioned as the primary parameter and the principle of parsimony was maintained for the supportive ML model.

### 2.7 Statistical and machine-learning analysis

Missing values were replaced with the arithmetic mean of the corresponding variable, computed on the analytic cohort (*N* = 89) before model fitting and without using the outcome; no participant was excluded because of missing data. Missingness was confined to selected laboratory variables (≤3.4% per variable), whereas the five parsimonious predictors (age, BMI, systolic blood pressure, HT duration, folate) were complete for all 89 participants; imputation therefore affected only the comparator models that included these laboratory variables. Continuous variables are summarized as mean ± standard deviation; categorical variables as count (percentage). Univariable group comparisons used the Mann–Whitney U test for continuous variables and Fisher exact test for categorical variables. Age-adjusted multivariable logistic regression provided odds ratios per inter-quartile range (IQR) increase for continuous predictors and per category for binary predictors.

The association between SCORE2 and CI was evaluated by categorical stratification (4 categories), χ^2^ for trend, Spearman rank correlation, continuous and categorical logistic regression (reference: Low SCORE2), and a multivariable model (SCORE2 + BMI). The discriminative ability of SCORE2 alone was evaluated by ROC analysis and AUC (area under the ROC curve); bootstrap (1,000 resamples) 95% confidence intervals were computed.

Cardiometabolic subgroup analysis stratified the cohort by clinically-validated thresholds: blood pressure (Normal <120/<80; Stage 1,120–139/80–89; Stage 2 ≥ 140/≥90, per 2017 ACC/AHA) (16), BMI (Normal <25; Overweight 25–29.9; Obese ≥30, per WHO) (19), hyperlipidemia (LDL ≥ 130 mg/dL or total cholesterol ≥200 mg/dL or current lipid-lowering therapy), smoking status (smoker / non-smoker), and fasting glucose category (Normal <100; Impaired fasting glucose 100–125; Diabetes ≥126 mg/dL or known diagnosis/treatment) (20). Two distinct hypertension classification systems were used in this study with non-overlapping purposes: cohort eligibility (HT vs. normotensive control) was determined exclusively by the 2018 ESC/ESH criteria (14) (office BP ≥ 140/90 mmHg or current antihypertensive therapy), as detailed in section 2.2; the 2017 ACC/AHA framework (21) was applied only as the analytic stratification of all 89 participants for the post-hoc dose–response evaluation, because its three-tier ordinal structure (Normal / Stage 1 / Stage 2) enables gradient analysis of CI prevalence across blood pressure strata, whereas the binary 2018 ESC/ESH definition does not. The 2017 ACC/AHA stratification therefore does not redefine group membership. The cumulative cardiometabolic risk score (0–4) was defined as: +1 for HT, +1 for obesity (BMI ≥ 30), +1 for hyperlipidemia, and +1 for impaired fasting glucose or diabetes. Trends across ordered categories were assessed by χ^2^ for trend; binary comparisons used Fisher exact test.

As supportive ML analyses, four candidate models were evaluated: L2-regularized logistic regression, L1-regularized LASSO logistic regression (22) (pre-specified parsimonious primary structure: age, BMI, SBP, HT duration, folate), Random Forest (23), and XGBoost (24). Model evaluation used 5-fold stratified cross-validation repeated 10 times. Predictor standardization was fitted within each training fold; discrimination was evaluated by AUC and area under the precision–recall curve (AUPRC) (25); calibration was evaluated by Brier score, visual calibration plots, and Hosmer-Lemeshow goodness-of-fit (26). Clinical utility was evaluated by Decision Curve Analysis (DCA) (27). Bootstrap (1.000 resamples) 95% CI were computed; bootstrap optimism correction (Harrell method, 200 bootstrap samples) was applied (28). Feature importance was evaluated with SHAP values (29). A bedside nomogram was derived from the parsimonious model as an exploratory tool, not for clinical deployment pending external validation. The parsimonious feature set (age, BMI, systolic blood pressure, HT duration, folate) was pre-specified rather than data-driven; missing values were limited (<4% overall, confined to selected laboratory variables) and were mean-imputed as described above; and model hyperparameters were fixed *a priori*, with no outcome-based tuning, to limit optimism at this sample size.

All analyses were performed in Python 3.12 (scikit-learn 1.5 (30), xgboost 3.2, shap 0.51, statsmodels 0.14).

## 3 Results

### 3.1 Participant characteristics

**Figure 1:**
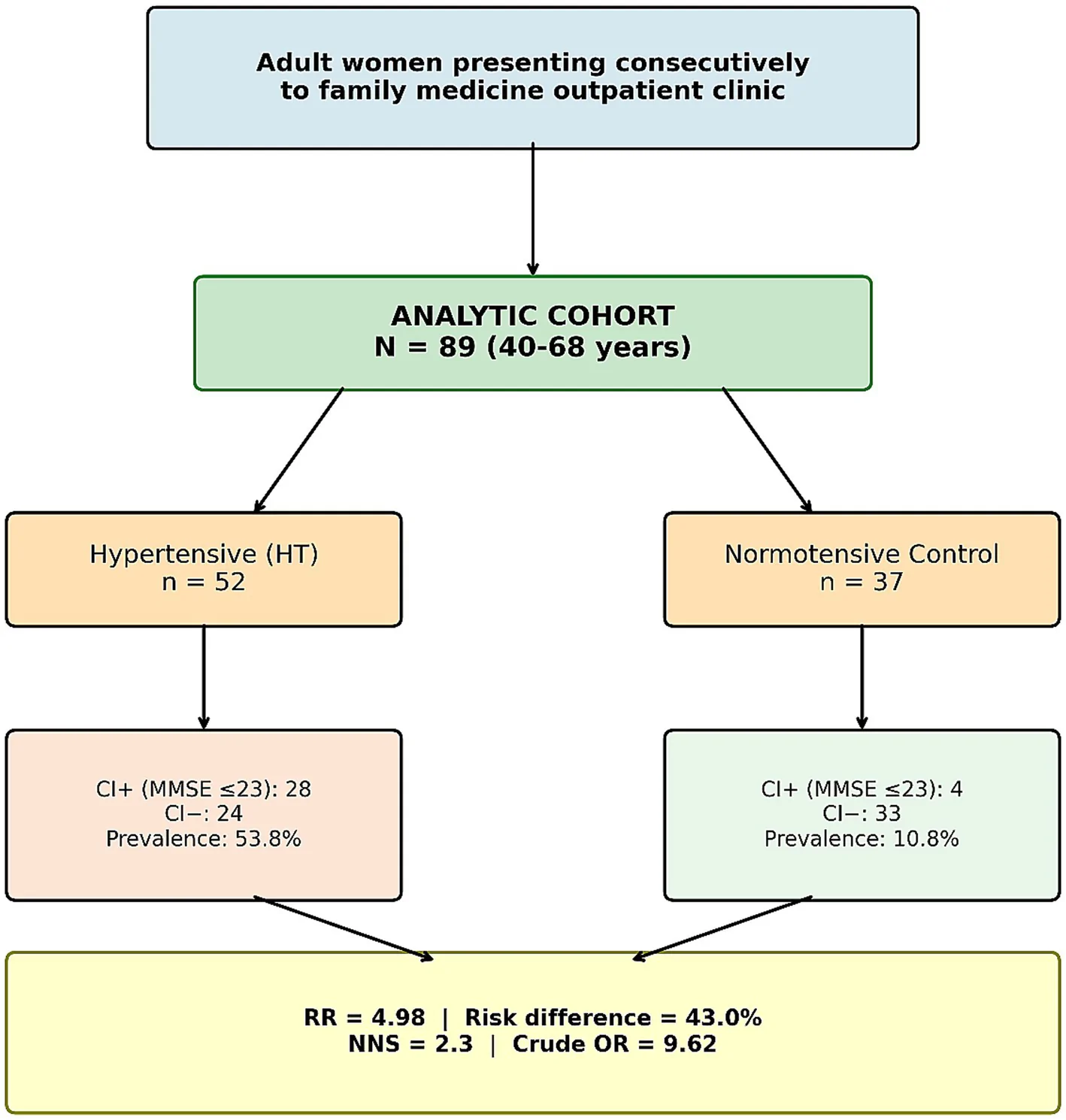
Participant flow diagram.

The analytic cohort comprised 89 women aged 40–68 years (52 HT, 37 normotensive controls; Table 1; Figure 1). Mean age was 50.2 ± 7.2 years. Cognitive impairment (CI; MMSE ≤23 by validated threshold) was identified in 32 participants (36.0%): 28/52 (53.8%) of HT participants and 4/37 (10.8%) of controls (Fisher exact *p* < 0.001). This corresponds to an unadjusted relative risk of 4.98 (95% CI 1.91–13.00), risk difference of 43.0 percentage points, number needed to screen (NNS) of 2.3, and crude odds ratio of 9.62 (95% CI 3.04–30.4). Women with CI were significantly older (53.1 ± 7.3 vs. 48.7 ± 6.7 years, *p* = 0.009), had higher BMI (32.0 ± 5.3 vs. 27.3 ± 5.3 kg/m^2^, *p* < 0.001), higher systolic and diastolic blood pressure (p < 0.001 and *p* = 0.001, respectively), and longer HT duration (6.7 ± 6.5 vs. 2.0 ± 3.5 years, p < 0.001). Lifestyle factors, lipid panel components, fasting glucose, and thyroid function tests did not differ significantly between groups. Folate was significantly lower in women with CI (6.47 ± 2.55 vs. 7.73 ± 2.89 ng/mL, *p* = 0.037); vitamin B12 was numerically lower in women with CI but did not reach statistical significance (224.8 ± 103.6 vs. 266.2 ± 124.9 pg/mL, *p* = 0.094).

**Table 1:** Baseline characteristics of the analytic cohort (*N* = 89) by cognitive-impairment status.

Characteristic	No CI (*n* = 57)	CI (*n* = 32)	*p*-value
Age, years	48.7 ± 6.7	53.1 ± 7.3	0.009
BMI, kg/m^2^	27.3 ± 5.3	32.0 ± 5.3	<0.001
Hypertension, *n* (%)	24 (42.1%)	28 (87.5%)	<0.001
HT duration, years	2.0 ± 3.5	6.7 ± 6.5	<0.001
SBP, mmHg	121.2 ± 14.7	139.5 ± 19.9	<0.001
DBP, mmHg	78.4 ± 8.5	84.8 ± 11.3	<0.001
Smoker, *n* (%)	9 (15.8%)	7 (21.9%)	0.568
Regular exercise, *n* (%)	12 (21.1%)	9 (28.1%)	0.198
Fasting glucose, mg/dL	91.1 ± 12.9	88.6 ± 13.8	0.399
Total cholesterol, mg/dL	208.6 ± 41.1	217.4 ± 52.1	0.499
Triglycerides, mg/dL	148.7 ± 91.5	178.4 ± 115.3	0.083
HDL, mg/dL	49.3 ± 15.4	47.5 ± 10.6	0.918
LDL, mg/dL	133.8 ± 36.1	135.9 ± 41.5	0.961
TSH, mIU/L	1.75 ± 1.21	1.96 ± 1.39	0.472
Vitamin B12, pg./mL	266.2 ± 124.9	224.8 ± 103.6	0.094
Folate, ng/mL	7.73 ± 2.89	6.47 ± 2.55	0.037

Continuous variables are mean ± SD with Mann–Whitney U *p*-values; categorical variables are count (%) with Fisher exact *p*-values. CI = MMSE ≤23 according to the validated Turkish threshold (16).

### 3.2 SCORE2 cardiovascular risk and cognitive impairment — primary finding

**Figure 2:**
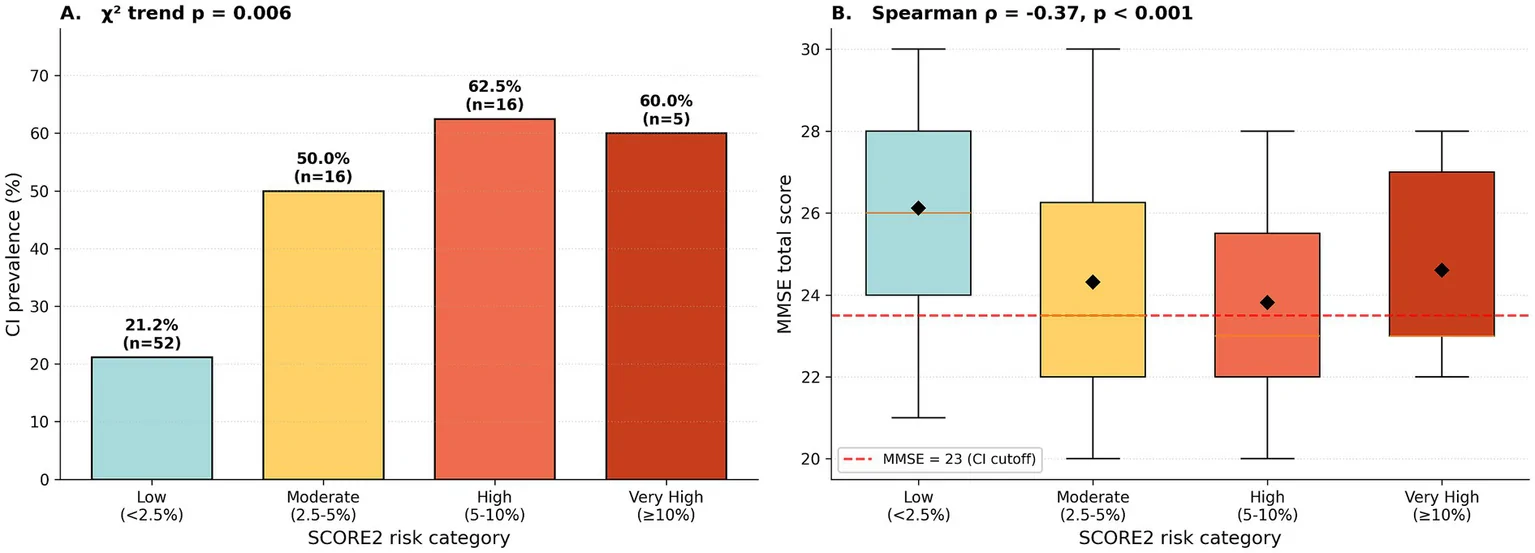
**(A)** Cognitive impairment prevalence by SCORE2 risk category, demonstrating a dose–response pattern (*χ*^2^ trend *p* = 0.006). **(B)** Distribution of MMSE total scores by SCORE2 category; the horizontal dashed red line indicates the MMSE ≤ 23 CI cut-off.

The primary finding of this study is the identification of a clear dose–response relationship between SCORE2 and CI. Mean SCORE2 was 3.07% ± 3.30%; 58.4% of the cohort fell into Low (<2.5%), 18.0% into Moderate (2.5–5%), 18.0% into High (5–10%), and 5.6% into Very High (≥10%) categories. CI prevalence increased monotonically across SCORE2 risk categories: 21.2% in Low; 50.0% in Moderate; 62.5% in High; 60.0% in Very High (Table 2; Figure 2A; χ^2^ trend *p* = 0.006; Spearman *ρ* = 0.37, *p* < 0.001). Mean MMSE total score declined from 26.1 ± 2.6 in the Low SCORE2 category to 23.8 ± 2.6 in the High category; the Spearman ρ between continuous SCORE2 and MMSE was −0.37 (*p* < 0.001) (Figure 2B).

**Table 2:** Association between SCORE2 cardiovascular risk categories and cognitive impairment (CI = MMSE ≤23).

SCORE2 risk category	*N*	No CI	CI+ (≤23)	CI Prev. (%)
Low (<2.5%)	52	41	11	21.2%
Moderate (2.5–5%)	16	8	8	50.0%
High (5–10%)	16	6	10	62.5%
Very high (≥10%)	5	2	3	60.0%
χ^2^ for trend	—	—	—	*p* = 0.006
Spearman ρ	—	—	—	0.37, *p* < 0.001

SCORE2 was computed with high-cardiovascular-risk-region coefficients (9, 17).

In univariable logistic regression, SCORE2 as a continuous variable was independently associated with CI: each 5% absolute increase in 10-year cardiovascular risk conferred OR 3.65 (95% CI 1.67–7.98, *p* = 0.001) for CI (Table 3). Categorical analysis (reference: Low SCORE2) showed a clear dose–response: Moderate OR 3.73 (1.14–12.19, *p* = 0.030); High OR 6.21 (1.85–20.86, *p* = 0.003); Very High OR 5.59 (0.83–37.72, *p* = 0.077; wide CI reflects the small subgroup, *n* = 5). Multivariable analysis including SCORE2 and BMI showed both remained independent predictors of CI (SCORE2 per 1% increase: OR 1.22, *p* = 0.012; BMI per kg/m^2^: OR 1.14, *p* = 0.007).

**Table 3:** Logistic regression models for the association between SCORE2 and cognitive impairment (continuous, categorical, and multivariable analysis with BMI).

Model/predictor	OR (95% CI)	*p-*value
SCORE2 (continuous, per 5% absolute risk increase)	3.65 (1.67–7.98)	0.001
SCORE2 categorical (vs Low)		
Moderate (2.5–5%)	3.73 (1.14–12.19)	0.034
High (5–10%)	6.21 (1.85–20.86)	0.003
Very High (≥10%)	5.59 (0.83–37.72)	0.077
Multivariable: SCORE2 + BMI		
SCORE2 (per 1% increase)	1.22 (1.05–1.43)	0.012
BMI (per 1 kg/m^2^ increase)	1.14 (1.04–1.25)	0.007

**Figure 3:**
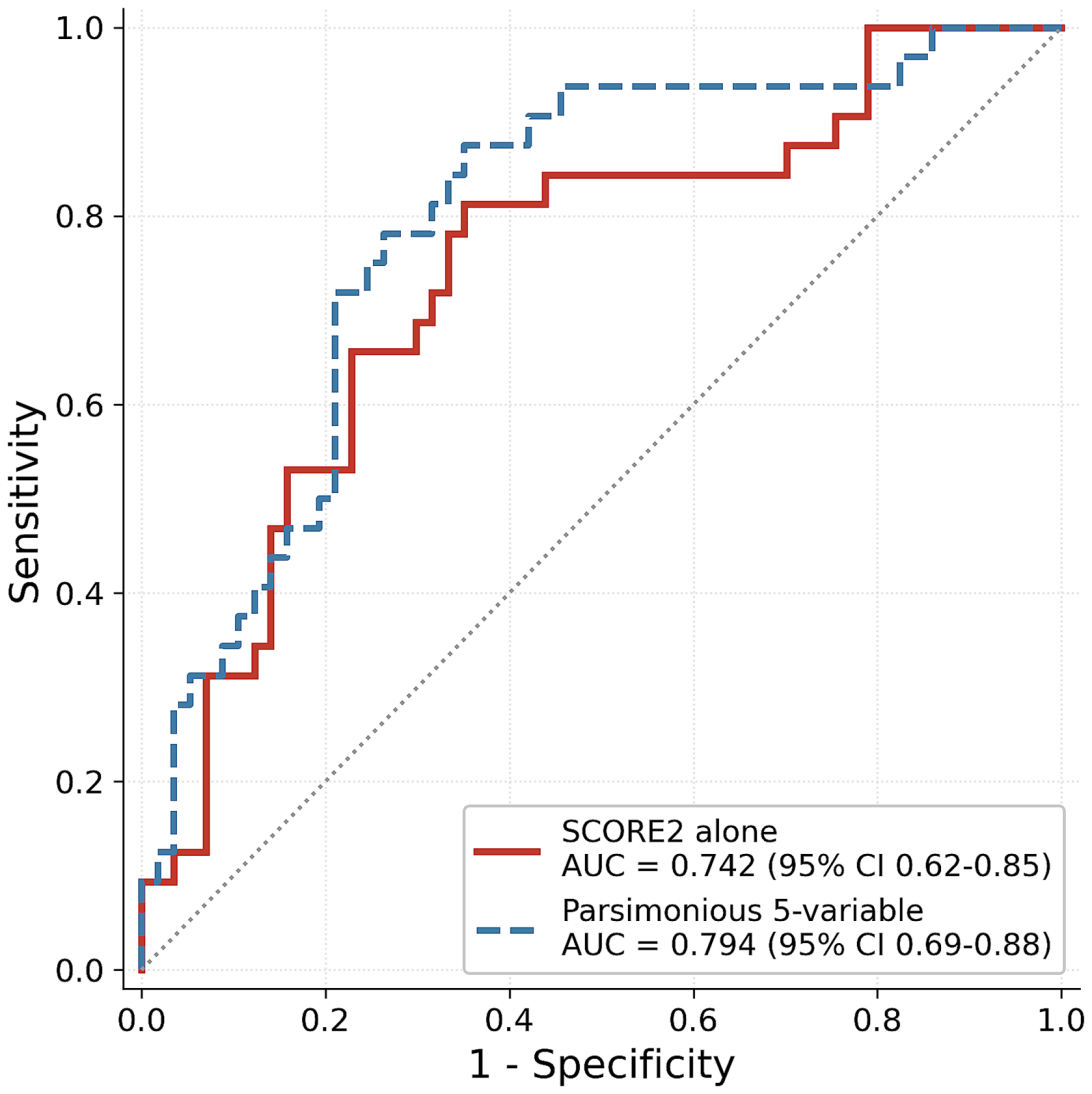
ROC curve for stand-alone discrimination of CI by SCORE2 (AUC = 0.742, 95% CI 0.62–0.85). For comparison, the ROC curve for the parsimonious 5-variable supportive model is also shown (dashed blue, AUC = 0.794, 95% CI 0.69–0.88).

As a stand-alone discriminative tool, SCORE2 discriminated CI with AUC 0.742 (95% CI 0.62–0.85) (Figure 3). This discriminative performance supports SCORE2’s positioning as a meaningful triage signal for CI.

One-carbon metabolism markers were also directionally associated with CI. Folate showed an inverse association with CI: a positive Spearman correlation with MMSE total score (*ρ* = 0.24, *p* = 0.023) and an age-adjusted univariable OR of 0.58 (95% CI 0.31–1.08, *p* = 0.084) per IQR decrement. Vitamin B12 correlated in the same direction with MMSE (ρ = 0.22, *p* = 0.043), with a borderline age-adjusted OR 0.70 (0.40–1.23; *p* = 0.213). Absolute deficiency prevalence was concordant: folate deficiency (<4 ng/mL) was 12.5% in women with CI versus 5.3% in those without; B12 deficiency (<200 pg/mL) was 43.8% versus 35.1%, respectively.

### 3.3 MMSE categorical distribution

Stratification of MMSE total score by Güngen 2002 categorical thresholds showed that CI was markedly skewed by HT status (Table 4): mild dementia-suspect scores (≤23) were approximately five-fold more frequent in HT participants than in controls (53.8% vs. 10.8%; χ^2^(2) = 17.4, *p* < 0.001).

**Table 4:** Categorical distribution of MMSE total score by hypertension status, using validated thresholds.

MMSE category	Control (*n* = 37)	HT (*n* = 52)	Total (*N* = 89)
Normal (≥27)	20 (54.1%)	15 (28.8%)	35 (39.3%)
Borderline (24–26)	13 (35.1%)	9 (17.3%)	22 (24.7%)
Mild dementia-suspect (≤23)	4 (10.8%)	28 (53.8%)	32 (36.0%)

### 3.4 Cardiometabolic subgroup analysis

CI prevalence showed a consistent gradient across clinically validated cardiometabolic strata (Table 5). For blood pressure, CI prevalence rose from 11.1% in normotensive women (BP < 120/<80), to 26.3% in Stage 1 hypertension (120–139/80–89), and to 60.6% in Stage 2 hypertension (≥140/≥90; trend *p* < 0.001). For BMI, prevalence was 13.8% in normal-weight, 23.8% in overweight, and 59.0% in obese women (trend p < 0.001). CI prevalence was similar between participants with and without hyperlipidemia (35.6% vs. 36.7%; *p* = 1.000); no significant difference was observed between smokers and non-smokers (43.8% vs. 34.2%; *p* = 0.568). The trend across fasting glucose categories did not reach statistical significance (Normal 40.0%; impaired fasting glucose 22.2%; diabetes 0%; trend *p* = 0.282).

**Table 5:** Cardiometabolic subgroup analysis: CI prevalence stratified by clinically validated thresholds.

Cardiometabolic subgroup	N	CI+ n (%)	*p-*value
Blood pressure category (2017 ACC/AHA)			*p* < 0.001^**1**^
Normal (<120/<80)	18	2 (11.1%)	
Stage 1 (120–139/80–89)	38	10 (26.3%)	
Stage 2 (≥140/≥90)	33	20 (60.6%)	
BMI category (WHO)			*p* < 0.001^**1**^
Normal weight (<25 kg/m^2^)	29	4 (13.8%)	
Overweight (25–29.9 kg/m^2^)	21	5 (23.8%)	
Obese (≥30 kg/m^2^)	39	23 (59.0%)	
Hyperlipidemia^2^			*p* = 1.000^**3**^
No	30	11 (36.7%)	
Yes	59	21 (35.6%)	
Smoking status			*p* = 0.568^**3**^
Non-smoker	73	25 (34.2%)	
Smoker	16	7 (43.8%)	
Fasting glucose category^4^			*p* = 0.282^**1**^
Normal (<100 mg/dL)	70	28 (40.0%)	
Impaired fasting glucose (100–125)	18	4 (22.2%)	
Diabetes (≥126 or known dx/treatment)	1	0 (0%)	
Cumulative cardiometabolic risk score^5^			*p* = 0.002^**1**^
0 risk factors	13	1 (7.7%)	
1 risk factor	17	4 (23.5%)	
2 risk factors	28	11 (39.3%)	
3 risk factors	28	14 (50.0%)	
4 risk factors	3	2 (66.7%)	

BP stratification follows the 2017 ACC/AHA framework (21) for analytic gradient evaluation; cohort enrolment (HT vs. control) was per 2018 ESC/ESH criteria (14) — See section 2.7. ^1^χ^2^ for trend across ordered categories. ^2^Hyperlipidemia: LDL ≥ 130 mg/dL or total cholesterol ≥200 mg/dL or current lipid-lowering therapy. ^3^Fisher exact test. ^4^Fasting glucose categories per ADA. ^5^Cumulative cardiometabolic risk score (0–4): One point each for HT, obesity (BMI ≥ 30), hyperlipidemia, and impaired fasting glucose/diabetes.

The pre-specified cumulative cardiometabolic risk score (HT + obesity + hyperlipidemia + impaired fasting glucose/diabetes; range 0–4) showed a clear dose–response pattern: CI prevalence was 7.7% in women with score 0, 23.5% with score 1, 39.3% with score 2, 50.0% with score 3, and 66.7% with score 4 (trend *p* = 0.002; Spearman *ρ* = 0.32, *p* = 0.002). This pattern indicates that cardiovascular risk is cumulatively associated with CI in a manner biologically consistent with the composite structure of SCORE2.

### 3.5 Age-adjusted multivariable associations

Age-adjusted multivariable logistic regression confirmed independent associations of cardiovascular components with CI (Table 6). Hypertension status increased the odds 7.6-fold (OR 7.63, 95% CI 2.28–25.51, *p* = 0.001); SBP per IQR (OR 4.32, *p* = 0.001), BMI per IQR (OR 4.18, *p* = 0.002), DBP per IQR (OR 2.94, *p* = 0.006), and HT duration per IQR (OR 2.71, *p* = 0.003) all remained independently associated. This age-adjusted multivariable evidence indicates that the association of SCORE2 with CI rests on the composite cardiovascular burden rather than a single component — making SCORE2 a more information-dense triage parameter than its individual components.

**Table 6:** Age-adjusted multivariable associations with cognitive impairment.

Predictor	Age-adjusted OR (95% CI)	*p*-value
Hypertension status (vs control)	7.63 (2.28–25.51)	0.001
SBP (per IQR)	4.32 (1.89–9.87)	0.001
BMI (per IQR)	4.18 (1.71–10.21)	0.002
DBP (per IQR)	2.94 (1.36–6.37)	0.006
HT duration (per IQR)	2.71 (1.42–5.18)	0.003

ORs are per IQR (interquartile range) for continuous predictors and per category for binary predictors.

### 3.6 Parsimonious ML model and clinical utility

**Figure 4:**
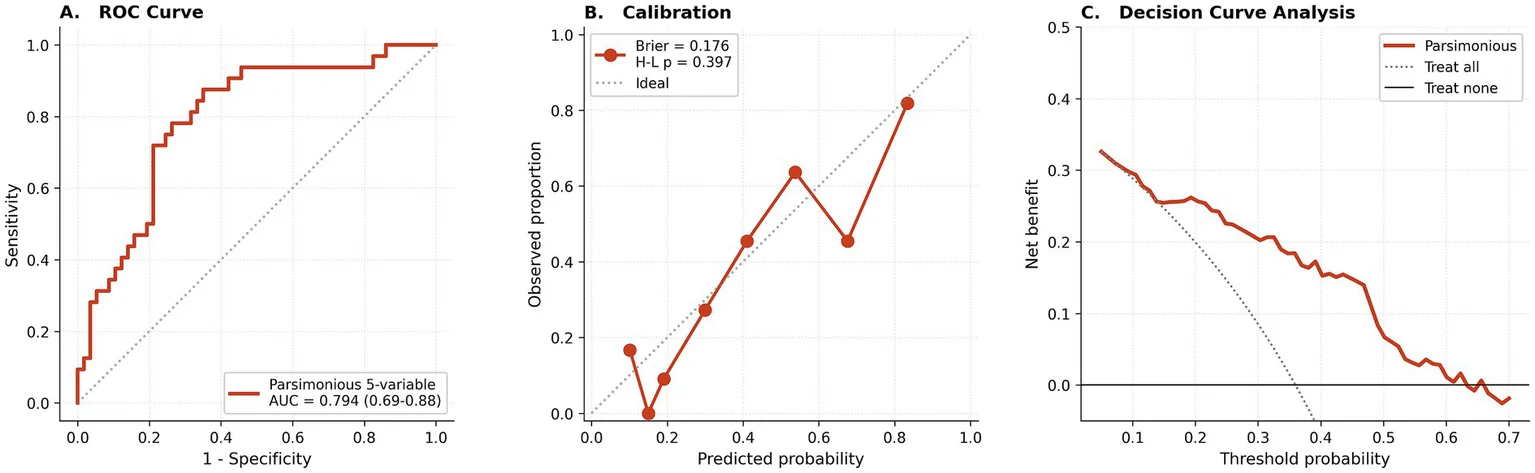
Performance of the parsimonious 5-variable supportive model. **(A)** ROC curve with bootstrap 95% CI. **(B)** Calibration plot with Brier score and Hosmer-Lemeshow goodness-of-fit. **(C)** Decision curve analysis across threshold probabilities.

To mechanistically support the SCORE2 signal, a parsimonious 5-variable (age, BMI, SBP, HT duration, folate) LASSO logistic regression model was developed. The model achieved a 10-times-repeated 5-fold cross-validated AUC of 0.804 ± 0.103 (single-fold OOF AUC 0.794; bootstrap 95% CI 0.689–0.884; Figure 4A), AUPRC 0.673, and Brier score 0.176 (Table 7). Bootstrap optimism correction yielded an apparent AUC of 0.842, optimism of 0.034, and optimism-corrected AUC of 0.808 — modest and well-quantified overfitting consistent with the small sample size. Hosmer-Lemeshow χ^2^ = 6.24 (*p* = 0.397) indicated adequate calibration (Figure 4B). Full LASSO (AUC 0.769), Random Forest (AUC 0.744), L2-regularized logistic regression (AUC 0.744), and XGBoost (AUC 0.715) did not outperform the parsimonious model (Table 7); this supports the principle of parsimony at this sample size. In an exploratory age-stratified comparison, CI prevalence was higher in women aged ≥60 (5/9, 55.6%) than <60 (27/80, 33.8%), and SCORE2 discrimination was preserved in both strata (AUC 0.80 and 0.75); however, only nine women were aged ≥60, precluding stable stratified model development, and age-specific risk structures (which rise sharply after 65) require larger cohorts.

**Table 7:** Discrimination and calibration of candidate models.

Model	AUC (mean ± SD)	Optimism-corr.	Brier
Age only (reference)	~0.66	—	—
SCORE2 alone	0.742	—	—
Parsimonious 5-variable (supportive)	0.804 ± 0.103	0.808	0.176
Full LASSO (18 variables)	0.769 ± 0.117	—	0.199
Random Forest (18 variables)	0.744 ± 0.128	—	0.193
L2-regularized logistic (18 variables)	0.744 ± 0.114		0.213
XGBoost (18 variables)	0.715 ± 0.124		0.209

Performance was evaluated using 5-fold stratified cross-validation repeated 10 times.

The incremental discrimination of the parsimonious model over SCORE2 alone was modest (ΔAUC +0.062). This improvement is small and of uncertain clinical significance, and does not by itself justify adopting the ML model in place of SCORE2 for triage. This clearly indicates that SCORE2 already contains most of the discriminative signal for CI and that an already-computed primary-care tool can be repurposed for cognitive risk. While the parsimonious model offers modest additional precision for clinics able to incorporate folate measurement and detailed HT-duration documentation, the main clinical message is that SCORE2 alone carries sufficient triage value.

**Figure 5:**
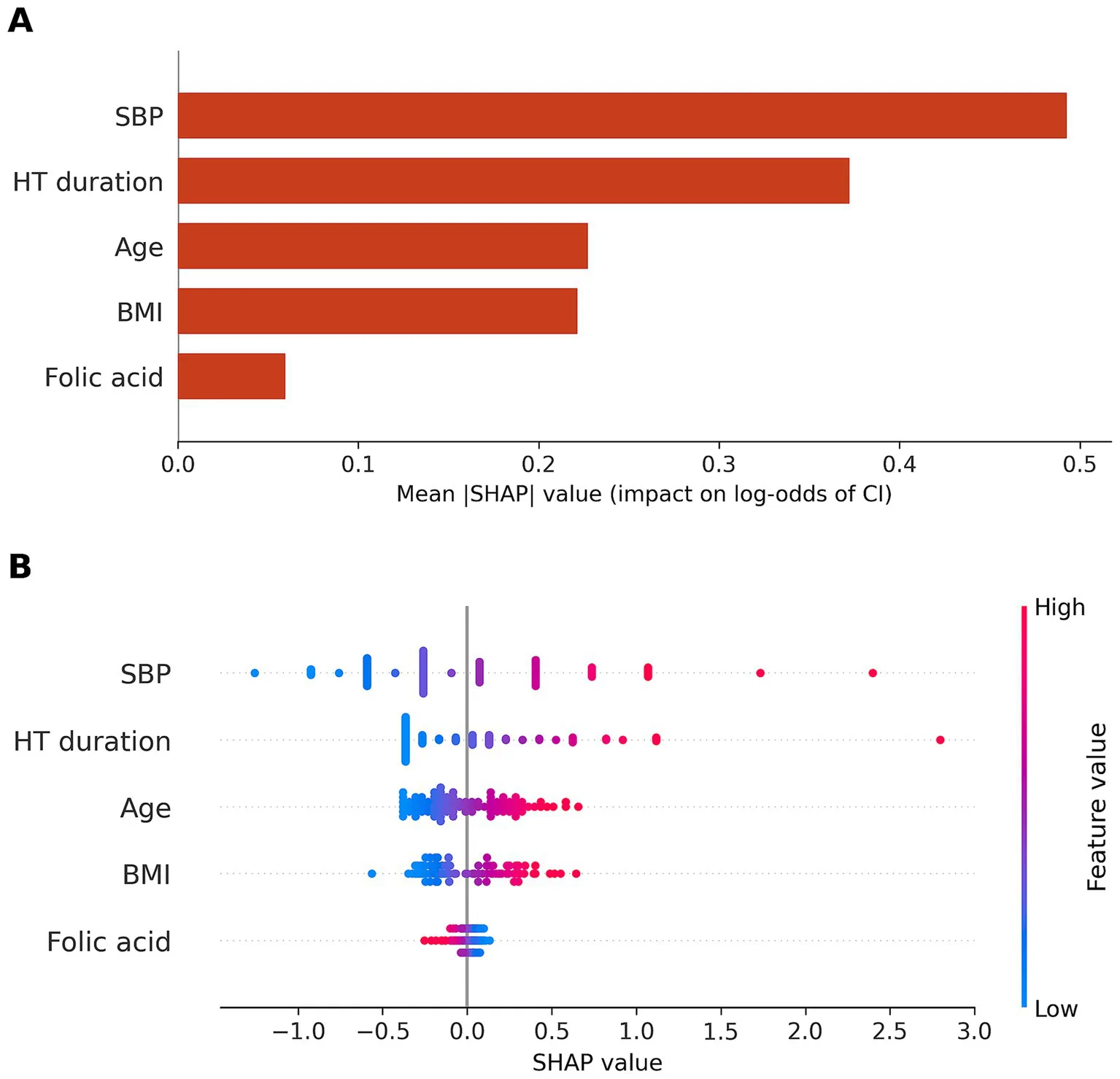
**(A)** Mean absolute SHAP values per predictor in the parsimonious model (feature importance ranking). **(B)** Distribution of individual SHAP values per predictor, colored by feature value (red = high; blue = low).

**Figure 6:**
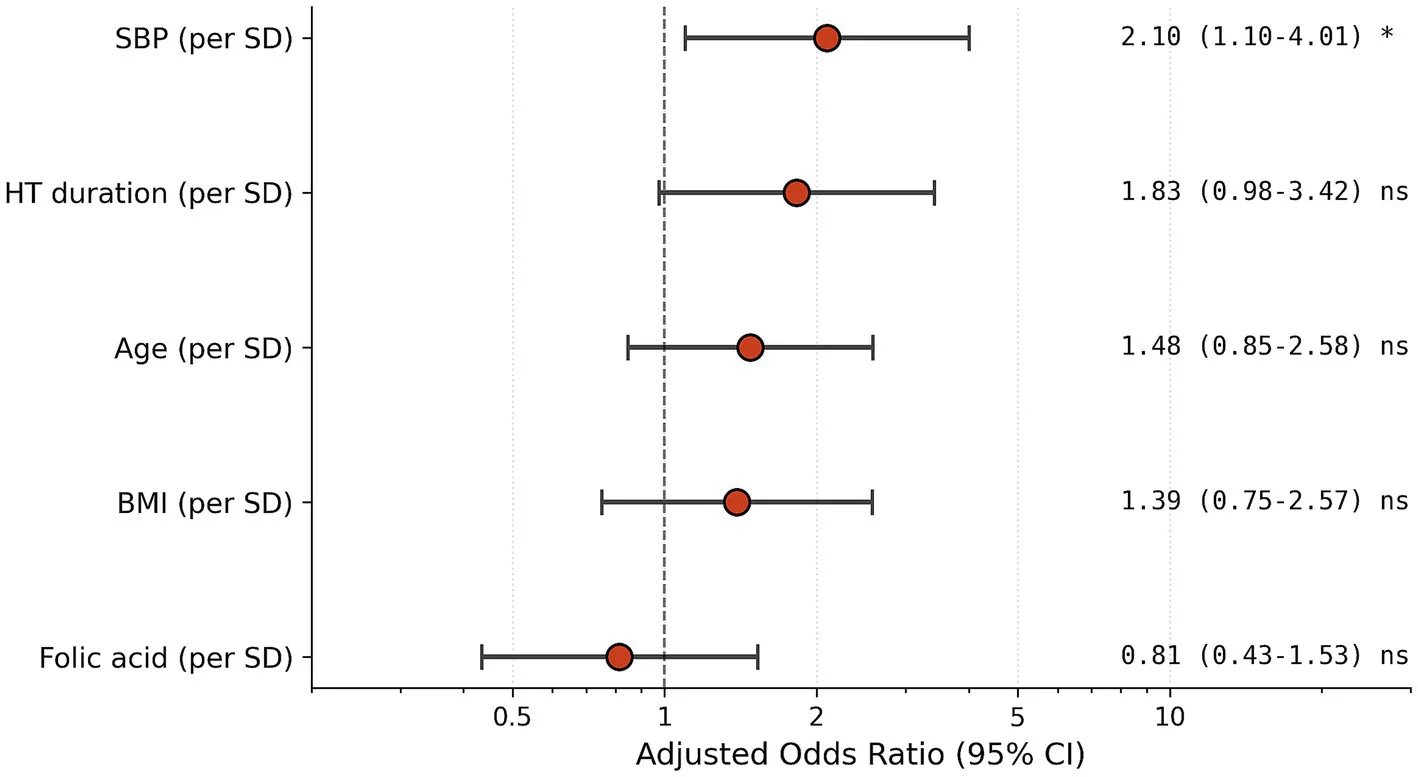
Forest plot of multivariable-adjusted ORs per standard deviation increase, jointly modeled in a single multivariable logistic regression.*p < 0.05; **p < 0.01; ***p < 0.001.

Decision Curve Analysis (Figure 4C) demonstrated that the parsimonious model provided positive net benefit across the threshold probability range of 0.10–0.55 versus both ‘treat-all’ and ‘treat-none’ reference strategies. SHAP analysis (Figure 5) confirmed that age, BMI, HT duration, SBP, and folate behaved directionally as mechanistically expected. The inverse relationship for folate (lower folate increasing CI risk) is consistent with the known impact of one-carbon metabolism and the homocysteine pathway on cognitive risk and contributes an independent layer of information to the model. The forest plot of standardized multivariable ORs (Figure 6) reflects the expected multicollinearity among HT-related predictors but shows that the joint predictive value of the panel is preserved.

**Figure 7:**
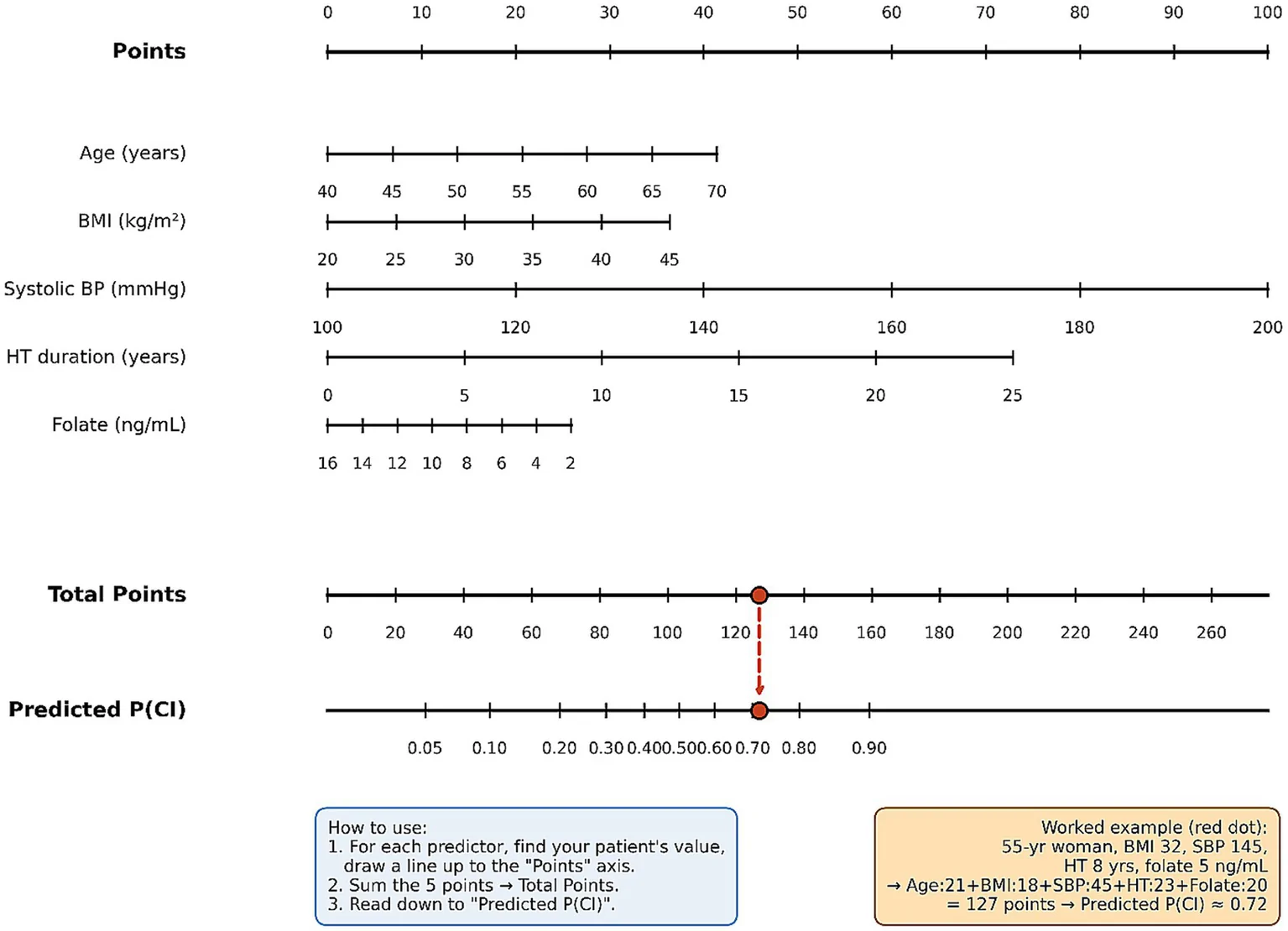
Bedside nomogram derived from the parsimonious 5-variable LASSO model for predicting cognitive impairment (MMSE ≤23). Lower folate yields higher points (inverse association); all other predictors: higher value → higher points. The red dot illustrates the worked example. Intended as an exploratory illustration only, not for clinical use pending external validation.

A bedside nomogram was derived from the parsimonious model as an exploratory tool (Figure 7). Clinicians can read the points associated with each predictor, sum them into a Total Points value, and directly read the predicted P(CI). Example: a 55-year-old woman with BMI 32 kg/m^2^, SBP 145 mmHg, HT duration 8 years, and folate 5 ng/mL accumulates 21 + 18 + 45 + 23 + 20 = 127 points, corresponding to a predicted P(CI) ≈ 0.72. Detailed scoring tables and a second worked example are provided in Supplementary Data Sheet 2.

## 4 Discussion

### 4.1 Principal findings

The principal finding of this study is that SCORE2 — already routinely calculated in primary care for cardiovascular risk — provides a clinically useful triage signal for cognitive impairment. CI prevalence across SCORE2 risk categories showed a clear dose–response pattern: from 21.2% in Low SCORE2 to 62.5% in High SCORE2 (an approximately three-fold increase; trend *p* = 0.006). Each 5% absolute increase in SCORE2 conferred OR 3.65 (1.67–7.98) for CI; SCORE2 alone discriminated CI with AUC 0.742 (0.62–0.85). This demonstrates that SCORE2 can serve not only as a cardiovascular event predictor, but also as a pragmatic proxy marker for cognitive risk.

Supportive analyses strengthen this primary signal. Overall CI prevalence was 36.0%; with HT 53.8% (NNS = 2.3), and a dose–response was observed across all evaluated cardiovascular/metabolic strata except smoking. The redefined cumulative cardiometabolic risk score (0–4) showed a gradient from 7.7 to 66.7% (*p* = 0.002). In age-adjusted multivariable analysis, all cardiovascular components (HT, SBP, BMI, DBP, HT duration) remained independently associated. The parsimonious 5-variable ML model performed well at AUC 0.804; however, its incremental discrimination over SCORE2 was modest (ΔAUC +0.062) — confirming that SCORE2 alone carries substantial cognitive-risk information.

### 4.2 Clinical implications of SCORE2 as a primary-care cognitive triage tool

Our findings have direct clinical implications. SCORE2 has been prospectively validated by the 2021 ESC guidelines (8, 9) and is already calculated as part of standard cardiovascular risk assessment in many primary-care settings. Our results indicate that women classified as high or very-high cardiovascular risk by SCORE2 warrant routine cognitive screening as part of comprehensive primary-care evaluation. This approach integrates two existing routine activities — cardiovascular risk stratification and cognitive screening — without requiring any new laboratory tests, equipment, or visit time.

As a practical recommendation, women with SCORE2 ≥ 5% may be considered for a brief cognitive screening test (MMSE or MoCA) as part of the visit. This threshold corresponds to the category in our cohort where CI prevalence exceeded 50%. This threshold is hypothesis-generating and must be prospectively externally validated before any clinical use. Once externally validated, this approach could enable systematic cognitive screening triage at no additional clinical resource cost.

For clinics requiring individualized risk communication beyond the SCORE2 triage step, a graphical nomogram was derived from the parsimonious model (Figure 7). The nomogram allows clinicians to (i) read the points associated with each of the five predictors (age, BMI, SBP, HT duration, folic acid), (ii) sum them into a Total Points value, and (iii) read the predicted P(CI) directly without an electronic device or software. Importantly, the nomogram does not replace but complements the SCORE2-based triage approach: SCORE2 provides a low-burden first-screen signal, while the nomogram offers individualized risk communication for clinics requiring additional precision. Detailed scoring tables and worked examples are provided in Supplementary Data Sheet 2. We clarify the intended pathway: SCORE2 serves as the low-cost first-stage triage; the confirmatory and diagnostic step for current cognitive impairment is a direct cognitive test (MMSE or MoCA), which the nomogram does not replace. The nomogram is an optional, exploratory risk-communication aid where a numeric probability is preferred, and its more natural application — estimating future incident CI — requires longitudinal validation.

### 4.3 Mechanistic plausibility

Our findings are strongly mechanistically consistent with the cardiovascular cognitive-impairment literature. High blood pressure and prolonged HT duration are associated with an increased risk of cognitive decline and dementia-related outcomes (6, 31). Higher BMI contributes via insulin resistance, systemic inflammation, and shared metabolic-vascular pathways (32, 33). Hyperlipidemia and glucose intolerance can contribute to vascular damage through atherosclerotic and metabolic mechanisms (34–37). Because SCORE2 integrates these components (age, blood pressure, total cholesterol, HDL, smoking) into a single composite score, an effect size larger than that of individual variables is biologically expected; this is consistent with the observed 62.5% prevalence.

The rise in CI prevalence from 7.7 to 66.7% across the cumulative cardiometabolic risk score (0–4; *p* = 0.002) supports an additive, biologically plausible relationship driven by composite cardiovascular burden rather than by a single dominant factor. This pattern reinforces the clinical meaningfulness of the parametric structure of SCORE2.

Beyond the vascular axis captured by SCORE2, one-carbon metabolism (folate–B12–homocysteine) constitutes a complementary, biologically distinct mechanism for cognitive risk. Folate and B12 deficiency lead to homocysteine accumulation, and hyperhomocysteinemia has been linked to endothelial dysfunction, oxidative stress, and cerebral small-vessel injury. The directionally inverse relationship of folate and B12 with CI in our cohort (folate *ρ* = 0.24, *p* = 0.023; B12 ρ = 0.22, *p* = 0.043) provides supportive evidence for this pathway. This finding mechanistically explains why folate carries independent predictive value in the parsimonious model beyond the SCORE2 components and suggests that routine measurement of serum folate and B12 may add a low-cost, actionable layer to CI triage in primary care.

### 4.4 Comparison with the literature

Our findings align with the established literature linking mid-life cardiovascular risk factors to late-life cognition (3, 6, 31, 33, 36, 37). Recent ML-based CI prediction studies in HT cohorts typically report results in the 0.70–0.85 AUC range (11, 31). The placement of SCORE2 alone at AUC 0.742 within this range supports the feasibility of repurposing an already-available tool for cognitive risk. Our SCORE2-alone discrimination (AUC 0.742) is consistent with published midlife/older-adult CI prediction models, which report C-statistics of approximately 0.71–0.81 (e.g., (38, 39)), although those models were developed in larger, longitudinal, and predominantly older cohorts; external validation in independent and public datasets remains a priority. To our knowledge, this is the first pragmatic feasibility study to directly evaluate the association of SCORE2 with CI and position it as a signal for primary-care triage. Because SCORE2 shares risk factors with other cardiometabolic conditions, it functions as a sensitive but non-specific first-stage triage rather than a diagnostic test; false positives are resolved at the confirmatory step — a brief cognitive test (MMSE/MoCA) — which, not SCORE2, establishes the outcome. Beyond the shared vascular axis, folate (one-carbon/homocysteine metabolism) contributes a mechanistically distinct, CI-relevant signal.

### 4.5 Strengths and limitations

Strengths include: positioning SCORE2, with its established prospective validation, as the primary parameter; an explicit feasibility framing aligned with TRIPOD+AI 2024 (12); use of routinely-collected primary-care data; preservation of the principle of parsimony through a pre-specified parsimonious model; comprehensive performance evaluation including calibration, Decision Curve Analysis, bootstrap optimism correction, and SHAP-based interpretability; anchoring the outcome to the validated SMMSE threshold (16); and provision of an exploratory bedside nomogram.

Several limitations should be noted. First, this is a single-center cross-sectional study; causal inference is not possible, and accordingly all SCORE2–CI findings represent cross-sectional associations rather than prediction or causation. Second, the cohort consists of women only; sex-specific risk-factor weights are well established (40). Because the cohort comprised only women from a single center, the findings may not generalize to men, to other ethnic groups, or to other primary-care and healthcare settings, and require external validation in more diverse populations before any extrapolation. Third, although all participants had completed at least primary education (≥5 years of schooling) as an inclusion criterion — which constrains the education-related variability that most affects MMSE performance — exact years of schooling were not recorded for finer adjustment, and socioeconomic status, depression, and menopausal status were likewise unmeasured; these represent potential residual confounders, and we cannot fully exclude that SCORE2 adds little beyond age and vascular burden. As stated in section 2.3, the MMSE-based binary outcome operates as a primary-care screening signal and not as a clinical CI diagnosis (41). The MMSE has limited sensitivity for mild cognitive impairment and is influenced by education; a more sensitive instrument such as the MoCA was not part of this routine primary-care dataset, and its use is recommended for confirmatory studies (Section 4.6). Fourth, an external validation cohort was not available; internal cross-validation and bootstrap optimism correction quantify within-sample overfitting but do not address out-of-distribution generalizability. Fifth, the cross-sectional design cannot capture incident cognitive decline. Sixth, given the limited sample (89 participants, 32 events; EPV ≈ 6.4), the machine-learning model, SHAP, decision-curve, and nomogram analyses are highly exploratory and hypothesis-generating, and several subgroup estimates rest on very small cells (Very-High SCORE2, n = 5; cumulative score 4, n = 3) with correspondingly wide uncertainty. Seventh, the cohort was essentially non-diabetic (one participant with diabetes); these findings therefore apply to non-diabetic women, and SCORE2-Diabetes would be the appropriate risk model for diabetic patients, whose cognitive-risk relationships we could not assess.

### 4.6 Roadmap to confirmatory studies

Confirmatory studies should: (i) recruit a multi-center primary-care cohort including male participants (target N ≥ 500); (ii) record exact years of education (beyond the primary-education inclusion threshold) and detailed socioeconomic data as essential confounders; (iii) supplement or replace MMSE with MoCA (42) or domain-specific neuropsychological tests; (iv) follow participants prospectively for ≥18 months to capture incident cognitive decline; (v) pre-specify external validation in an independent center; and (vi) test the screening yield of a SCORE2-based triage threshold (e.g., SCORE2 ≥ 5%) in a randomized implementation trial; and (vii) evaluate the effect of folate/B12 supplementation on cognitive outcomes in women at high SCORE2 risk in randomized trials.

## 5 Conclusion

SCORE2 — already routinely calculated in primary care — provides a clinically useful triage signal for screening-defined cognitive impairment at no additional resource cost; each 5% absolute increase in SCORE2 approximately triples the odds of CI, and SCORE2 alone discriminates CI with AUC 0.742. Women at high cardiovascular risk (particularly those with SCORE2 ≥ 5%) may benefit from routine cognitive screening as part of integrated primary-care evaluation. The supportive parsimonious ML model mechanistically complements the SCORE2 signal; the independent inverse association of folate reflects the contribution of one-carbon metabolism beyond the vascular axis and offers modest incremental precision. A bedside nomogram is provided as an exploratory tool, not for clinical use pending external validation. As a single-center exploratory feasibility study, findings are intended to provide a basis for multi-center prospective validation rather than for direct clinical deployment.

## Data Availability

Anonymized analytical data may be obtained from the corresponding author upon reasonable request, subject to approval by the Tekirdağ Namık Kemal University Clinical Research Ethics Committee and applicable confidentiality regulations.

## References

[1] GBD 2019 Dementia Forecasting Collaborators. Estimation of the global prevalence of dementia in 2019 and forecasted prevalence in 2050: an analysis for the Global Burden of Disease Study 2019. Lancet Public Health. (2022) 7:e105–25. doi: 10.1016/S2468-2667(21)00249-8, 34998485 PMC8810394

[2] Livingston G Huntley J Sommerlad A Ames D Ballard C Banerjee S . Dementia prevention, intervention, and care: 2020 report of the lancet commission. Lancet. (2020) 396:413–46. doi: 10.1016/S0140-6736(20)30367-6, 32738937 PMC7392084

[3] Iadecola C Yaffe K Biller J Bratzke LC Faraci FM Gorelick PB . Impact of hypertension on cognitive function: a scientific statement from the American Heart Association. Hypertension. (2016) 68:e67–94. doi: 10.1161/HYP.0000000000000053, 27977393 PMC5361411

[4] Iadecola C. The pathobiology of vascular dementia. Neuron. (2013) 80:844–66. doi: 10.1016/j.neuron.2013.10.008, 24267647 PMC3842016

[5] Debette S Markus HS. The clinical importance of white matter hyperintensities on brain magnetic resonance imaging: systematic review and meta-analysis. BMJ. (2010) 341:c3666. doi: 10.1136/bmj.c3666, 20660506 PMC2910261

[6] SPRINT MIND Investigators for the SPRINT Research Group; Williamson JD Pajewski NM Auchus AP Bryan RN Chelune G Cheung AK . Effect of intensive vs standard blood pressure control on probable dementia: a randomized clinical trial. JAMA. (2019) 321:553–61. doi: 10.1001/jama.2018.21442, 30688979 PMC6439590

[7] de la Torre JC. Cardiovascular risk factors promote brain hypoperfusion leading to cognitive decline and dementia. Cardiovasc Psychiatry Neurol. (2012) 2012:367516. doi: 10.1155/2012/367516, 23243502 PMC3518077

[8] Visseren FLJ Mach F Smulders YM Carballo D Koskinas KC Bäck M . 2021 ESC guidelines on cardiovascular disease prevention in clinical practice. Eur Heart J. (2021) 42:3227–337. doi: 10.1093/eurheartj/ehab484, 34458905

[9] SCORE2 working group and ESC Cardiovascular risk collaboration. SCORE2 risk prediction algorithms: new models to estimate 10-year risk of cardiovascular disease in Europe. Eur Heart J. (2021) 42:2439–54. doi: 10.1093/eurheartj/ehab309, 34120177 PMC8248998

[10] Goerdten J Čukić I Danso SO Carrière I Muniz-Terrera G. Statistical methods for dementia risk prediction and recommendations for future work: a systematic review. Alzheimers Dement (N Y). (2019) 5:563–9. doi: 10.1016/j.trci.2019.08.001, 31646170 PMC6804431

[11] Hou X-H Feng L Zhang C Cao X-P Tan L Yu J-T. Models for predicting risk of dementia: a systematic review. J Neurol Neurosurg Psychiatry. (2019) 90:373–9. doi: 10.1136/jnnp-2018-318212, 29954871

[12] Collins GS Moons KGM Dhiman P Riley RD Beam AL Calster BV . TRIPOD+AI statement: updated guidance for reporting clinical prediction models that use regression or machine learning methods. BMJ. (2024) 385:e078378. doi: 10.1136/bmj-2023-078378, 38626948 PMC11019967

[13] Vandenbroucke JP von Elm E Altman DG Gøtzsche PC Mulrow CD Pocock SJ . Strengthening the reporting of observational studies in epidemiology (STROBE): explanation and elaboration. Ann Intern Med. (2007) 147:W-163–94. doi: 10.7326/0003-4819-147-8-200710160-00010-w1, 17938389

[14] Williams B Mancia G Spiering W Agabiti Rosei E Azizi M Burnier M . 2018 ESC/ESH guidelines for the management of arterial hypertension. Eur Heart J. (2018) 39:3021–104. doi: 10.1093/eurheartj/ehy339, 30165516

[15] Folstein MF Folstein SE McHugh PR. “Mini-mental state”. A practical method for grading the cognitive state of patients for the clinician. J Psychiatr Res. (1975) 12:189–98. doi: 10.1016/0022-3956(75)90026-6, 1202204

[16] Güngen C Ertan T Eker E Yaşar R Engin F. Reliability and validity of the standardized Mini mental state examination in the diagnosis of mild dementia in Turkish population. Turk Psikiyatri Derg. (2002) 13:273–81. 12794644

[17] Hageman SHJ Kaptoge S Gynnild MN Holtrop J Pennells L McEvoy JW . Using SCORE2 with a risk chart or online calculator: impact on model performance, treatment eligibility, and cardiovascular disease prevention. Eur Heart J Qual Care Clin Outcomes. (2025) 12:213–20. doi: 10.1093/ehjqcco/qcaf122, 41071935 PMC13016803

[18] Riley RD Snell KI Ensor J Burke DL Harrell FE Moons KG . Minimum sample size for developing a multivariable prediction model: PART II - binary and time-to-event outcomes. Stat Med. (2019) 38:1276–96. doi: 10.1002/sim.7992 30357870 PMC6519266

[19] World Health Organization. Obesity: preventing and managing the global epidemic. Report of a WHO consultation. World Health Organ Tech Rep Ser. (2000) 894:i–xii, 1–253. 11234459

[20] American Diabetes Association Professional Practice Committee. Diagnosis and classification of diabetes: standards of care in diabetes-2024. Diabetes Care. (2024) 47:S20–42. doi: 10.2337/dc24-S002 38078589 PMC10725812

[21] Whelton PK Carey RM Aronow WS Casey DE Collins KJ Dennison Himmelfarb C . 2017 ACC/AHA/AAPA/ABC/ACPM/AGS/APhA/ASH/ASPC/NMA/PCNA guideline for the prevention, detection, evaluation, and management of high blood pressure in adults: a report of the American College of Cardiology/American Heart Association task force on clinical practice guidelines. Hypertension. (2017) 71:e13–e115. doi: 10.1161/HYP.0000000000000065, 29133356

[22] Tibshirani R. Regression shrinkage and selection via the lasso. J R Stat Soc Ser B Stat Methodol. (1996) 58:267–88. doi: 10.1111/j.2517-6161.1996.tb02080.x

[23] Breiman L. Random forests. Mach Learn. (2001) 45:5–32. doi: 10.1023/A:1010933404324

[24] Chen T Guestrin C. XGBoost: A Scalable Tree Boosting System Proceedings of the 22nd ACM SIGKDD International Conference on Knowledge Discovery and Data Mining. New York, NY, USA: Association for Computing Machinery (2016). p. 785–94.

[25] Steyerberg EW Vickers AJ Cook NR Gerds T Gonen M Obuchowski N . Assessing the performance of prediction models: a framework for traditional and novel measures. Epidemiology. (2010) 21:128–38. doi: 10.1097/EDE.0b013e3181c30fb2, 20010215 PMC3575184

[26] Van Calster B McLernon DJ van Smeden M Wynants L Steyerberg EW Topic Group ‘Evaluating diagnostic tests and prediction models’ of the STRATOS initiative. Calibration: the Achilles heel of predictive analytics. BMC Med. (2019) 17:230. doi: 10.1186/s12916-019-1466-7, 31842878 PMC6912996

[27] Vickers AJ Elkin EB. Decision curve analysis: a novel method for evaluating prediction models. Med Decis Mak. (2006) 26:565–74. doi: 10.1177/0272989X06295361, 17099194 PMC2577036

[28] Harrell FE Lee KL Mark DB. Multivariable prognostic models: issues in developing models, evaluating assumptions and adequacy, and measuring and reducing errors. Stat Med. (1996) 15:361–87. doi: 10.1002/(SICI)1097-0258(19960229)15:4<361::AID-SIM168>3.0.CO;2-4, 8668867

[29] Lundberg S. M. Lee S-I. A Unified Approach to Interpreting Model Predictions. Advances in Neural Information Processing Systems. Curran Associates, Inc. (2017). Available online at: https://papers.nips.cc/paper_files/paper/2017/hash/8a20a8621978632d76c43dfd28b67767-Abstract.html

[30] Pedregosa F Varoquaux G Gramfort A Michel V Thirion B Grisel O . Scikit-learn: machine learning in Python. J Mach Learn Res. (2011) 12, 2825–30.

[31] Gottesman RF Albert MS Alonso A Coker LH Coresh J Davis SM . Associations between midlife vascular risk factors and 25-year incident dementia in the atherosclerosis risk in communities (ARIC) cohort. JAMA Neurol. (2017) 74:1246–54. doi: 10.1001/jamaneurol.2017.1658, 28783817 PMC5710244

[32] Pase MP Beiser A Enserro D Xanthakis V Aparicio H Satizabal CL . Association of Ideal Cardiovascular Health with Vascular Brain Injury and Incident Dementia. Stroke. (2016) 47:1201–6. doi: 10.1161/strokeaha.115.012608, 27073239 PMC5006676

[33] Pase MP Davis-Plourde K Himali JJ Satizabal CL Aparicio H Seshadri S . Vascular risk at younger ages most strongly associates with current and future brain volume. Neurology. (2018) 91:e1479–86. doi: 10.1212/wnl.0000000000006360, 30232248 PMC6202941

[34] Sabia S Fayosse A Dumurgier J Schnitzler A Empana J-P Ebmeier KP . Association of ideal cardiovascular health at age 50 with incidence of dementia: 25 year follow-up of Whitehall II cohort study. BMJ. (2019) 366:l4414. doi: 10.1136/bmj.l4414, 31391187 PMC6664261

[35] Gorelick PB Scuteri A Black SE Decarli C Greenberg SM Iadecola C . Vascular contributions to cognitive impairment and dementia: a statement for healthcare professionals from the american heart association/american stroke association. Stroke. (2011) 42:2672–713. doi: 10.1161/STR.0b013e3182299496, 21778438 PMC3778669

[36] Kivipelto M Ngandu T Laatikainen T Winblad B Soininen H Tuomilehto J. Risk score for the prediction of dementia risk in 20 years among middle aged people: a longitudinal, population-based study. Lancet Neurol. (2006) 5:735–41. doi: 10.1016/S1474-4422(06)70537-3, 16914401

[37] Yaffe K Vittinghoff E Pletcher MJ Hoang TD Launer LJ Whitmer R . Early adult to midlife cardiovascular risk factors and cognitive function. Circulation. (2014) 129:1560–7. doi: 10.1161/CIRCULATIONAHA.113.004798, 24687777 PMC4700881

[38] Zhang X Fan H Guo C Li Y Han X Xu Y . Establishment of a mild cognitive impairment risk model in middle-aged and older adults: a longitudinal study. Neurol Sci. (2024) 45:4269–78. doi: 10.1007/s10072-024-07536-2, 38642322

[39] Huang M Gao X Zhao R Dong C Gu Z Gao J. Development and validation of a nomogram for predicting mild cognitive impairment in middle-aged and elderly people. Asian J Psychiatr. (2022) 75:103224. doi: 10.1016/j.ajp.2022.103224, 35870309

[40] Ferretti MT Iulita MF Cavedo E Chiesa PA Schumacher Dimech A Santuccione Chadha A . Sex differences in Alzheimer disease - the gateway to precision medicine. Nat Rev Neurol. (2018) 14:457–69. doi: 10.1038/s41582-018-0032-9, 29985474

[41] Petersen RC Lopez O Armstrong MJ Getchius TSD Ganguli M Gloss D . Practice guideline update summary: mild cognitive impairment [RETIRED]: report of the guideline development, dissemination, and implementation Subcommittee of the American Academy of neurology. Neurology. (2018) 90:126–35. doi: 10.1212/WNL.0000000000004826, 29282327 PMC5772157

[42] Nasreddine ZS Phillips NA Bédirian V Charbonneau S Whitehead V Collin I . The Montreal cognitive assessment, MoCA: a brief screening tool for mild cognitive impairment. J Am Geriatr Soc. (2005) 53:695–9. doi: 10.1111/j.1532-5415.2005.53221.x, 15817019

