# Supplementary material for: SCORE2 cardiovascular risk score for cognitive impairment in women aged 40 years and above: a pragmatic triage signal in primary care — a single-center family medicine feasibility study with machine learning support
Source: Front Med (Lausanne). 2026 Aug 19;13:1884887. doi: 10.3389/fmed.2026.1884887 (PMC13534097; doi:10.3389/fmed.2026.1884887)
Supplement: Supplementary file 1 [file Table_1.DOCX]

**Supplementary Material**

**Manuscript:** *SCORE2 Cardiovascular Risk Score for Cognitive Impairment in Women Aged 40 Years and Above: A Pragmatic Triage Signal in Primary Care — A Single-Center Family Medicine Feasibility Study with Machine Learning Support*

# **Supplementary Data Sheet 1 — TRIPOD+AI 2024 Reporting Checklist**

**Reference:** *Prepared according to the TRIPOD+AI reporting guideline (Collins et al., 2024).*

This checklist documents how each TRIPOD+AI 2024 reporting item is addressed in the main manuscript. Section numbers refer to the structure of the main manuscript.

**TRIPOD+AI Checklist — Items 1–24**

| **Item** | **Section/Topic** | **Checklist Item** | **Page/Section in Manuscript** |
| --- | --- | --- | --- |
| **1** | **Title** | Identify the study as developing or evaluating a multivariable prediction model, the target population, and the outcome to be predicted. | *Title page — identifies the study as a feasibility prediction study targeting CI in women aged ≥40.* |
| **2** | **Abstract** | Provide a structured summary of objectives, methods, results, and conclusions. | *Abstract — Background, Methods, Results, Conclusions, Keywords sections present.* |
| **3a** | **Background and rationale** | Explain the medical context (including whether diagnostic or prognostic) and rationale for developing or evaluating the prediction model, including references to existing models. | *Section 1 (Introduction), paragraphs 1–3.* |
| **3b** | **Objectives** | Specify the objectives, including whether the study describes the development, validation, or update of a prediction model. | *Section 1 (Introduction), final paragraph: feasibility study with primary aim of characterizing SCORE2-CI association.* |
| **4a** | **Source of data** | Describe the study design or source of data (e.g., randomized trial, cohort, registry data), separately for development and validation datasets. | *Section 2.1 (Study Design and Setting): single-center, cross-sectional, exploratory feasibility study at Department of Family Medicine.* |
| **4b** | **Eligibility criteria** | Specify the key elements of the study setting (e.g., primary care, secondary care, general population) including dates, follow-up period, and key elements of the inclusion and exclusion criteria. | *Section 2.2 (Participants): inclusion and exclusion criteria detailed.* |
| **5a** | **Outcome** | Clearly define the outcome that is predicted, including how and when assessed, with a rationale. | *Section 2.3 (Outcome Definition): MMSE total score ≤23 according to the validated Turkish cut-off (Güngen et al., 2002).* |
| **5b** | **Outcome assessment** | Report any actions to blind assessment of the outcome to be predicted. | *Section 2.3: MMSE administered face-to-face by a single trained family medicine resident, blinded to laboratory results and blood pressure values.* |
| **6a** | **Predictors** | Clearly define all predictors used in developing the multivariable prediction model, including how and when they were measured. | *Section 2.4 (Predictor Variables): 18 routinely-collected primary-care variables specified, all measured at baseline visit.* |
| **6b** | **Predictor assessment** | Report any actions to blind assessment of predictors for the outcome and other predictors. | *Section 2.4: laboratory analyses performed in central institutional laboratory using standardized commercial kits; predictors measured before MMSE administration.* |
| **7** | **Sample size** | Explain how the study size was arrived at. | *Section 2.6 (Sample-Size Considerations): events-per-variable (EPV) ratio was approximately 6.4 for the five-predictor model, consistent with an exploratory feasibility design (Riley et al.).* |
| **8** | **Missing data** | Describe how missing data were handled (e.g., complete-case analysis, single imputation, multiple imputation) with details of any imputation method. | *Section 2.7: KNN imputation (k=5) applied within each cross-validation fold; few missing values across predictors.* |
| **9a** | **Model development** | Describe how predictors were handled in the analyses, including approach for selection. | *Section 2.7: predictors standardized within each training fold; LASSO regularization for variable selection in supportive ML model.* |
| **9b** | **Model evaluation** | Specify the type of model, all model-building procedures (including any predictor selection), and method for internal validation. | *Section 2.7: four candidate models compared (L2 logistic, LASSO logistic, Random Forest, regularized XGBoost); 10-times-repeated 5-fold stratified cross-validation for internal validation.* |
| **10** | **Risk groups** | If a risk-grouping system was developed, explain how it was created and any rationale. | *Section 2.5 (SCORE2 Computation): risk categories per 2021 ESC guidelines (Low <2.5%, Moderate 2.5–5%, High 5–10%, Very High ≥10%).* |
| **11** | **Software/code** | Identify the software (with version) and packages used to perform the analysis. | *Section 2.7, final paragraph: Python 3.12, scikit-learn 1.5, xgboost 3.2, shap 0.51, statsmodels 0.14.* |
| **12** | **Funding** | Provide information on funding sources for the model development study. | *Funding statement: this research received no external funding.* |
| **13** | **Conflicts of interest** | Declare any conflicts of interest and financial disclosures for all authors. | *Conflict of Interest statement: authors declare no conflicts of interest.* |
| **14** | **Data sharing** | Provide details of where data may be obtained for replication purposes. | *Data Availability Statement: de-identified analytic data and reproducible code are available from the corresponding author upon reasonable request, subject to institutional approval and applicable ethical/privacy regulations.* |
| **15** | **Code sharing** | Provide details of where the model code may be obtained. | *Section 2.7 last sentence and Data Availability Statement: reproducible code is available from the corresponding author upon reasonable request, subject to institutional approval.* |
| **16** | **Ethical approval** | Provide details of ethical approval and informed consent. | *Section 2.1 (Study Design and Setting) and Ethics Statement.* |
| **17a** | **Participants — flow** | Provide a flow chart showing the number of participants and the number of cases (or events) at each stage of model development. | *Figure 1 (Participant flow diagram): N=89 analytic cohort, HT n=52, Control n=37, with CI counts.* |
| **17b** | **Participants — characteristics** | Report participant characteristics (basic demographics, clinical features, available predictors), including the number of participants with missing data for predictors and outcome. | *Table 1 and Section 3.1 (Participant Characteristics).* |
| **18** | **Model development** | Report the unadjusted and adjusted associations between each candidate predictor and outcome. | *Tables 1, 3, 6 (univariable, age-adjusted, and SCORE2-based logistic regression results).* |
| **19** | **Model performance** | Report performance measures (with confidence intervals) for the prediction model, including discrimination and calibration. | *Section 3.6, Table 7, Figure 4: AUC, AUPRC, Brier, Hosmer-Lemeshow, calibration plot, decision curve analysis.* |
| **20** | **Model specification** | Present the final prediction model (i.e., all regression coefficients, and model intercept or baseline risk) with details on how to apply. | *Figure 7 (bedside nomogram); Supplementary Data Sheet 2 provides full coefficient values, scoring tables, and worked examples.* |
| **21a** | **Interpretation** | Give an overall interpretation of the results, considering objectives, limitations, and results from similar studies. | *Section 4.1 (Principal Findings) and Section 4.4 (Comparison with the Literature).* |
| **21b** | **Limitations** | Discuss any limitations of the study (such as nonrepresentative sample, few events per predictor, missing data). | *Section 4.5 (Strengths and Limitations): single-center, cross-sectional, female-only, no education data, no external validation cohort.* |
| **22** | **Implications** | Discuss the potential clinical use of the model and implications for future research. | *Section 4.2 (Clinical Implications), Section 4.6 (Roadmap to Confirmatory Studies), Section 4.7 (Conclusions).* |
| **23** | **Supplementary information** | Provide information about availability of supplementary resources (e.g., study protocol, web calculators, datasets). | *Supplementary Data Sheet 1 (this TRIPOD+AI checklist); Supplementary Data Sheet 2 (detailed nomogram scoring tables and worked examples); Supplementary Data Sheet 3 (STROBE checklist).* |
| **24** | **Registration** | Provide information about study registration, where applicable. | *Not applicable — this is an exploratory feasibility study; not a registered clinical trial.* |
