# Supplementary material for: SCORE2 cardiovascular risk score for cognitive impairment in women aged 40 years and above: a pragmatic triage signal in primary care — a single-center family medicine feasibility study with machine learning support
Source: Front Med (Lausanne). 2026 Aug 19;13:1884887. doi: 10.3389/fmed.2026.1884887 (PMC13534097; doi:10.3389/fmed.2026.1884887)
Supplement: Supplementary file 2 [file Table_2.DOCX]

**Supplementary Material**

**Manuscript:** *SCORE2 Cardiovascular Risk Score for Cognitive Impairment in Women Aged 40 Years and Above: A Pragmatic Triage Signal in Primary Care — A Single-Center Family Medicine Feasibility Study with Machine Learning Support*

# **Supplementary Data Sheet 2 — Bedside Nomogram: Detailed Scoring Tables and Worked Examples**

## **S2.1 Underlying Model Specification**

The bedside nomogram (Figure 7 in the main manuscript) is derived from a parsimonious 5-variable logistic regression model fitted on the analytic cohort (N=89). The full mathematical specification is:

*logit(P(CI)) = β0 + β1·Age + β2·BMI + β3·SBP + β4·HTduration + β5·Folate*

**Logistic Regression Coefficients**

| **Predictor** | **Coefficient (β)** | **Interpretation** |
| --- | --- | --- |
| Intercept (β₀) | **-10.1621** | *Baseline log-odds at all reference values* |
| Age (years) | **+0.0543** | *Each 1-year increase → +5.6% odds of CI* |
| BMI (kg/m²) | **+0.0573** | *Each 1 kg/m² increase → +5.9% odds of CI* |
| SBP (mmHg) | **+0.0394** | *Each 1 mmHg increase → +4.0% odds of CI* |
| HT duration (years) | **+0.1147** | *Each 1-year increase → +12.1% odds of CI* |
| Folate (ng/mL) | **-0.0728** | *Each 1 ng/mL increase → −7.0% odds of CI (inverse association)* |

*All coefficients fitted with standard maximum-likelihood logistic regression on the full analytic cohort (N=89). For internal validation and bootstrap-corrected performance, see Section 3.6 and Table 7 of the main manuscript.*

## **S2.2 Per-Predictor Scoring Tables**

For nomogram use, each predictor value is mapped to a 'point' value. The scoring system normalizes each predictor's contribution to a 0–100 scale relative to the predictor with the largest absolute effect (HT duration). The maximum total points possible is approximately 277.

**Age (years)**

| **Age value** | **Points** |
| --- | --- |
| 40 | **0.0** |
| 42 | **2.8** |
| 44 | **5.5** |
| 46 | **8.3** |
| 48 | **11.0** |
| 50 | **13.8** |
| 52 | **16.6** |
| 54 | **19.3** |
| 56 | **22.1** |
| 58 | **24.8** |
| 60 | **27.6** |
| 62 | **30.4** |
| 64 | **33.1** |
| 66 | **35.9** |
| 68 | **38.6** |
| 70 | **41.4** |

**BMI (kg/m²)**

| **BMI value** | **Points** |
| --- | --- |
| 20 | **0.0** |
| 22 | **2.9** |
| 24 | **5.8** |
| 26 | **8.7** |
| 28 | **11.6** |
| 30 | **14.6** |
| 32 | **17.5** |
| 34 | **20.4** |
| 36 | **23.3** |
| 38 | **26.2** |
| 40 | **29.1** |
| 42 | **32.0** |
| 44 | **34.9** |

**Systolic BP (mmHg)**

| **Systolic value** | **Points** |
| --- | --- |
| 100 | **0.0** |
| 110 | **10.0** |
| 120 | **20.0** |
| 130 | **30.0** |
| 140 | **40.0** |
| 150 | **50.0** |
| 160 | **60.0** |
| 170 | **70.0** |
| 180 | **80.0** |
| 190 | **90.0** |
| 200 | **100.0** |

**HT duration (years)**

| **HT value** | **Points** |
| --- | --- |
| 0 | **0.0** |
| 2 | **5.8** |
| 4 | **11.7** |
| 6 | **17.5** |
| 8 | **23.3** |
| 10 | **29.2** |
| 12 | **35.0** |
| 14 | **40.8** |
| 16 | **46.6** |
| 18 | **52.5** |
| 20 | **58.3** |
| 22 | **64.1** |
| 24 | **70.0** |

**Folate (ng/mL)**

| **Folate value** | **Points** |
| --- | --- |
| 2 | **25.9** |
| 3 | **24.0** |
| 4 | **22.2** |
| 5 | **20.3** |
| 6 | **18.5** |
| 7 | **16.6** |
| 8 | **14.8** |
| 9 | **12.9** |
| 10 | **11.1** |
| 11 | **9.2** |
| 12 | **7.4** |
| 13 | **5.5** |
| 14 | **3.7** |
| 15 | **1.8** |
| 16 | **0.0** |

## **S2.3 Total Points → Predicted P(CI) Lookup Table**

After summing the points from all five predictors, locate the closest value below to estimate P(CI):

| **Total Points** | **P(CI)** | **P(CI) — %** | **Risk Category** |
| --- | --- | --- | --- |
| 0 | 0.017 | **1.7%** | **Low** |
| 10 | 0.025 | **2.5%** | **Low** |
| 20 | 0.036 | **3.6%** | **Low** |
| 30 | 0.053 | **5.3%** | **Low** |
| 40 | 0.076 | **7.6%** | **Low** |
| 50 | 0.109 | **10.9%** | **Moderate** |
| 60 | 0.153 | **15.3%** | **Moderate** |
| 70 | 0.211 | **21.1%** | **Moderate** |
| 80 | 0.284 | **28.4%** | **Moderate** |
| 90 | 0.371 | **37.1%** | **High** |
| 100 | 0.466 | **46.6%** | **High** |
| 110 | 0.564 | **56.4%** | **High** |
| 120 | 0.657 | **65.7%** | **Very High** |
| 130 | 0.740 | **74.0%** | **Very High** |
| 140 | 0.808 | **80.8%** | **Very High** |
| 150 | 0.862 | **86.2%** | **Very High** |
| 160 | 0.903 | **90.3%** | **Very High** |
| 170 | 0.932 | **93.2%** | **Very High** |
| 180 | 0.953 | **95.3%** | **Very High** |
| 190 | 0.968 | **96.8%** | **Very High** |
| 200 | 0.978 | **97.8%** | **Very High** |
| 210 | 0.985 | **98.5%** | **Very High** |
| 220 | 0.990 | **99.0%** | **Very High** |
| 230 | 0.993 | **99.3%** | **Very High** |
| 240 | 0.995 | **99.5%** | **Very High** |
| 250 | 0.997 | **99.7%** | **Very High** |

*Risk categories shown above are clinical heuristics for triage discussion only, not validated cut-offs. Clinical decisions should consider the full context including age, comorbidities, and patient preference.*

## **S2.4 Worked Examples**

The following four worked examples illustrate how the nomogram is applied across a range of risk profiles encountered in primary care.

**Example 1 — Low-risk profile**

*A 45-year-old normotensive woman with normal BMI, optimal blood pressure, and adequate folate.*

| **Predictor** | **Patient Value** | **Points** |
| --- | --- | --- |
| Age (years) | 45 | **6.9** |
| BMI (kg/m²) | 24 | **5.8** |
| Systolic BP (mmHg) | 115 | **15.0** |
| HT duration (years) | 0 | **0.0** |
| Folate (ng/mL) | 10 | **11.1** |
| **TOTAL POINTS** | — | **38.8** |

**Predicted P(CI) = 0.073 (7.3%) — Low risk category**

**Example 2 — Moderate-risk profile**

*A 52-year-old woman with stage 1 hypertension (5 years), overweight, with mid-range folate.*

| **Predictor** | **Patient Value** | **Points** |
| --- | --- | --- |
| Age (years) | 52 | **16.6** |
| BMI (kg/m²) | 29 | **13.1** |
| Systolic BP (mmHg) | 135 | **35.0** |
| HT duration (years) | 5 | **14.6** |
| Folate (ng/mL) | 7 | **16.6** |
| **TOTAL POINTS** | — | **95.9** |

**Predicted P(CI) = 0.426 (42.6%) — High risk category**

**Example 3 — High-risk profile**

*A 55-year-old hypertensive woman (8 years) with class I obesity, elevated SBP, and low folate.*

| **Predictor** | **Patient Value** | **Points** |
| --- | --- | --- |
| Age (years) | 55 | **20.7** |
| BMI (kg/m²) | 32 | **17.5** |
| Systolic BP (mmHg) | 145 | **45.0** |
| HT duration (years) | 8 | **23.3** |
| Folate (ng/mL) | 5 | **20.3** |
| **TOTAL POINTS** | — | **126.8** |

**Predicted P(CI) = 0.715 (71.5%) — Very High risk category**

**Example 4 — Very high-risk profile**

*A 62-year-old woman with longstanding hypertension (15 years), class II obesity, severe SBP elevation, and folate deficiency.*

| **Predictor** | **Patient Value** | **Points** |
| --- | --- | --- |
| Age (years) | 62 | **30.4** |
| BMI (kg/m²) | 38 | **26.2** |
| Systolic BP (mmHg) | 165 | **65.0** |
| HT duration (years) | 15 | **43.7** |
| Folate (ng/mL) | 3 | **24.0** |
| **TOTAL POINTS** | — | **189.3** |

**Predicted P(CI) = 0.967 (96.7%) — Very High-risk category**

## **S2.5 Clinical Interpretation Guidance**

The nomogram is intended as a primary-care triage aid. Suggested clinical interpretation:

| **Predicted P(CI)** | **Risk Category** | **Suggested Action** |
| --- | --- | --- |
| **<10%** | **Low** | Routine cardiovascular care; no specific cognitive testing required at this visit. Re-assess at next annual visit. |
| **10–30%** | **Moderate** | Consider brief cognitive screening (MMSE or MoCA) at next visit; address modifiable risk factors. |
| **30–60%** | **High** | Recommend cognitive screening (MMSE/MoCA) at this visit; intensify cardiovascular risk-factor management; consider folate/B12 measurement. |
| **>60%** | **Very High** | Cognitive screening (MMSE/MoCA) at this visit; if MMSE ≤23, refer for comprehensive neuropsychological assessment; aggressive cardiovascular and metabolic risk-factor management. |
