# Supplementary material for: SCORE2 cardiovascular risk score for cognitive impairment in women aged 40 years and above: a pragmatic triage signal in primary care — a single-center family medicine feasibility study with machine learning support
Source: Front Med (Lausanne). 2026 Aug 19;13:1884887. doi: 10.3389/fmed.2026.1884887 (PMC13534097; doi:10.3389/fmed.2026.1884887)
Supplement: Supplementary file 3 [file Table_3.DOCX]

**Supplementary Material**

**Manuscript:** *SCORE2 Cardiovascular Risk Score for Cognitive Impairment in Women Aged 40 Years and Above: A Pragmatic Triage Signal in Primary Care — A Single-Center Family Medicine Feasibility Study with Machine Learning Support*

# **Supplementary Data Sheet 3 — STROBE Statement: Checklist for Cross-Sectional Studies**

**Source and Reference**

This checklist is based on the official STROBE Statement Checklist for Cross-Sectional Studies, available from the STROBE Initiative website. The structure, item numbering, and recommendations follow the STROBE Statement verbatim; the right-hand column documents how each item is addressed in the present manuscript.

**Official source:** https://www.strobe-statement.org/checklists/ — checklist version: 'STROBE Checklist: cross-sectional studies' (downloadable as PDF or Word).

This document reproduces the 22 items applicable to cross-sectional studies and maps each item to its location in the present manuscript. Section numbers refer to the structure of the main manuscript.

**STROBE Checklist — Items 1–22 (Cross-Sectional Studies)**

| **Section/Topic** | **Item** | **Recommendation** | **Location in this Manuscript** |
| --- | --- | --- | --- |
| **Title and abstract** | **1** | (a) Indicate the study's design with a commonly used term in the title or the abstract; (b) Provide in the abstract an informative and balanced summary of what was done and what was found. | *Title identifies study as 'feasibility study' (cross-sectional). Abstract is structured (Background, Methods, Results, Conclusions) and includes design, key results, and conclusions.* |
| **Background/rationale** | **2** | Explain the scientific background and rationale for the investigation being reported. | *Section 1 (Introduction), paragraphs 1–3: rationale for studying SCORE2 as a cognitive triage signal.* |
| **Objectives** | **3** | State specific objectives, including any prespecified hypotheses. | *Section 1 (Introduction), final paragraph: primary aim (characterize SCORE2-CI association) and secondary aim (parsimonious ML model).* |
| **Study design** | **4** | Present key elements of study design early in the paper. | *Section 2.1 (Study Design and Setting): single-center, cross-sectional, exploratory feasibility study.* |
| **Setting** | **5** | Describe the setting, locations, and relevant dates, including periods of recruitment, exposure, follow-up, and data collection. | *Section 2.1: Department of Family Medicine, Tekirdağ Namık Kemal University.* |
| **Participants** | **6** | (a) Give the eligibility criteria, and the sources and methods of selection of participants. | *Section 2.2 (Participants): consecutive women aged 40–68 attending family medicine outpatient clinic; HT and control inclusion/exclusion criteria detailed.* |
| **Variables** | **7** | Clearly define all outcomes, exposures, predictors, potential confounders, and effect modifiers. Give diagnostic criteria, if applicable. | *Section 2.3 (Outcome Definition): MMSE ≤23 (Güngen et al., 2002). Section 2.4 (Predictor Variables): 18 routine primary-care variables.* |
| **Data sources/measurement** | **8** | For each variable of interest, give sources of data and details of methods of assessment (measurement). Describe comparability of assessment methods if there is more than one group. | *Section 2.3: blinded MMSE administration. Section 2.4: standardized commercial kits in central institutional laboratory. Same protocol applied to both HT and control groups.* |
| **Bias** | **9** | Describe any efforts to address potential sources of bias. | *Section 2.3: outcome assessor blinded to laboratory and BP values. Section 2.4: predictors measured before MMSE. Section 2.7: target leakage avoided by excluding MMSE sub-domains from predictors.* |
| **Study size** | **10** | Explain how the study size was arrived at. | *Section 2.6 (Sample-Size Considerations): EPV ratio approximately 6.4 for the five-predictor model.* |
| **Quantitative variables** | **11** | Explain how quantitative variables were handled in the analyses. If applicable, describe which groupings were chosen and why. | *Section 2.7: continuous variables standardized within each CV fold. Categorical groupings per validated thresholds: BP (2017 ACC/AHA), BMI (WHO), glucose (ADA), SCORE2 (2021 ESC).* |
| **Statistical methods** | **12** | (a) Describe all statistical methods, including those used to control for confounding; (b) Describe any methods used to examine subgroups and interactions; (c) Explain how missing data were addressed; (d) If applicable, describe analytical methods taking account of sampling strategy; (e) Describe any sensitivity analyses. | *Section 2.7 (Statistical and ML Analysis): comprehensive description of Mann-Whitney, Fisher exact, logistic regression, χ² trend, ROC, bootstrap, calibration, DCA, KNN imputation; subgroup analysis defined; sensitivity analyses via repeated CV.* |
| **Participants — flow** | **13** | (a) Report numbers of individuals at each stage of study (e.g., numbers potentially eligible, examined for eligibility, confirmed eligible, included in the study, completing follow-up, and analysed); (b) Give reasons for non-participation at each stage; (c) Consider use of a flow diagram. | *Figure 1 (Participant flow diagram): N=89 analytic cohort with breakdown by group and CI status.* |
| **Descriptive data** | **14** | (a) Give characteristics of study participants (e.g., demographic, clinical, social) and information on exposures and potential confounders; (b) Indicate number of participants with missing data for each variable of interest. | *Table 1 and Section 3.1 (Participant Characteristics): demographic, clinical, lipid, glucose, thyroid, B12, folate values for CI+ and CI− groups.* |
| **Outcome data** | **15** | Report numbers of outcome events or summary measures. | *Section 3.1 and Figure 1: 32/89 (36.0%) CI+ overall; 28/52 (53.8%) HT vs 4/37 (10.8%) controls.* |
| **Main results** | **16** | (a) Give unadjusted estimates and, if applicable, confounder-adjusted estimates and their precision (e.g., 95% confidence interval). Make clear which confounders were adjusted for and why they were included; (b) Report category boundaries when continuous variables were categorized; (c) If relevant, consider translating estimates of relative risk into absolute risk for a meaningful time period. | *Section 3.2: SCORE2 categorical and continuous OR with 95% CI, dose-response. Sections 3.3–3.5: cardiometabolic subgroup analysis with category boundaries; age-adjusted multivariable ORs in Table 6. Absolute risk reported (53.8% vs 10.8%, NNS=2.3).* |
| **Other analyses** | **17** | Report other analyses done — e.g., analyses of subgroups and interactions, and sensitivity analyses. | *Section 3.4 (Cardiometabolic subgroup analysis); Section 3.6 (Parsimonious ML model with internal validation, calibration, DCA, SHAP).* |
| **Key results** | **18** | Summarize key results with reference to study objectives. | *Section 4.1 (Principal Findings): key SCORE2-CI dose-response, AUC 0.742, parsimonious model performance.* |
| **Limitations** | **19** | Discuss limitations of the study, taking into account sources of potential bias or imprecision. Discuss both direction and magnitude of any potential bias. | *Section 4.5 (Strengths and Limitations): single-center, cross-sectional, female-only, no education data, no external validation, limited sample size.* |
| **Interpretation** | **20** | Give a cautious overall interpretation of results considering objectives, limitations, multiplicity of analyses, results from similar studies, and other relevant evidence. | *Sections 4.1–4.4: cautious interpretation referencing literature (ARIC, Whitehall II, AHA statements) and acknowledging multi-test analyses.* |
| **Generalisability** | **21** | Discuss the generalisability (external validity) of the study results. | *Sections 4.5 and 4.6: female-only single-center cohort limits generalisability; external validation in multi-center cohorts including male participants is identified as essential next step.* |
| **Funding** | **22** | Give the source of funding and the role of the funders for the present study and, if applicable, for the original study on which the present article is based. | *Funding statement: this research received no external funding.* |
